# Microsatellite Markers in Olives (*Olea europaea* L.): Utility in the Cataloging of Germplasm, Food Authenticity and Traceability Studies

**DOI:** 10.3390/foods10081907

**Published:** 2021-08-17

**Authors:** Shambhavi Yadav, Joana Carvalho, Isabel Trujillo, Marta Prado

**Affiliations:** 1 Genetics and Tree Improvement Division, Forest Research Institute, P.O. New Forest, Dehradun 248001, India; 2 Food Quality and Safety Research Group, International Iberian Nanotechnology Laboratory (INL), 4715-330 Braga, Portugal; joana.rr.carvalho@gmail.com (J.C.); marta.prado@inl.int (M.P.); 3 Department of Analytical Chemistry, Nutrition and Food Science, Campus Vida, College of Pharmacy/School of Veterinary Sciences, University of Santiago de Compostela, E-15782 Santiago de Compostela, Spain; 4 Excellence Unit of Maria de Maeztu, Department of Agronomy, Rabanales Campus, International Campus of Excellence on Agrofood (ceiA3), University of Córdoba, 14014 Córdoba, Spain

**Keywords:** authentication, cultivar identification, *Olea europaea*, olive oil, simple sequence repeats, traceability, table olive

## Abstract

The olive fruit, a symbol of Mediterranean diets, is a rich source of antioxidants and oleic acid (55–83%). Olive genetic resources, including cultivated olives (cultivars), wild olives as well as related subspecies, are distributed widely across the Mediterranean region and other countries. Certain cultivars have a high commercial demand and economical value due to the differentiating organoleptic characteristics. This might result in economically motivated fraudulent practices and adulteration. Hence, tools to ensure the authenticity of constituent olive cultivars are crucial, and this can be achieved accurately through DNA-based methods. The present review outlines the applications of microsatellite markers, one of the most extensively used types of molecular markers in olive species, particularly referring to the use of these DNA-based markers in cataloging the vast olive germplasm, leading to identification and authentication of the cultivars. Emphasis has been given on the need to adopt a uniform platform where global molecular information pertaining to the details of available markers, cultivar-specific genotyping profiles (their synonyms or homonyms) and the comparative profiles of oil and reference leaf samples is accessible to researchers. The challenges of working with microsatellite markers and efforts underway, mainly advancements in genotyping methods which can be effectively incorporated in olive oil varietal testing, are also provided. Such efforts will pave the way for the development of more robust microsatellite marker-based olive agri-food authentication platforms.

## 1. Introduction

The olive tree has been cultivated for approximately 6000 years in Mediterranean countries, where 95% of olive germplasm is located. Its habitat is determined by the Mediterranean climate, and it stands as the most highly cultivated fruit crop among temperate crops in the world. According to data published by International Olive Council (IOC) (www.international.oliveoil.org (accessed on 10 February 2021)), in the last 25 years, olive oil production and consumption has increased by 1 million tons. The olive crop is mainly located in the Mediterranean Basin (the leading producers being Spain, Italy and Greece). Moreover, the olive is also a crop under increasing cultivation in non-traditional countries such as Argentina, Australia, Chile, China, Japan and the United States.

Both olive oil and fruits have been found to be a rich source of antioxidants and various other secondary metabolites (phenolics, carotenoids, tocopherols, anthocyanins and oleosides). Olive oil in particular has an unique lipid fatty acid composition and health benefits such as defense against chronic degenerative diseases, and reduced cardiovascular risks are attributed to the consumption of olive oils and table olives [1,2,3]. The increased demand of nutritionally superior olive oil such as extra virgin olive oil (EVOO) and virgin olive oil (VOO) and table olives has also led to increased adulteration of premium quality oils and fruits. Hence, regulations and certifications such as protected designation of origin (PDO) and protected geographical indication (PGI) (EC Regulation no. 510/2006) have been laid out to check product authenticity and traceability.

The exchange of germplasm in ancient times and increased commerce among olive growing nations has established complex genetic relationships among different olive gene pools [4]. The cultivation of cultivars in new climatic conditions and the adoption of local names for new introduced material have led to confusion in the denominations of varieties [5,6]. More than 1200 cultivars of olive spread across the Mediterranean region, with around 600 olive cultivars under cultivation in Italy itself, have been described in the olive germplasm database [7]. The characterization and recognition of many other cultivars and ancient and wild forms is still an ongoing process, and several studies have been undertaken in this direction using morphological as well as molecular tools [8]. Germplasm banks have been established to ensure ex situ conservation of olive genetic resources, and emphasis is being given to the use of microsatellite markers or simple sequence repeats (SSRs) as tools to better inventory these valuable repositories. Molecular characterization or genotypic profiling of available germplasm will not only provide unique identification keys but also help in the development of molecular authentication platforms, wherein these cultivars, wild forms or related species can easily and accurately be identified. Ever since being developed, SSRs or microsatellites are among the most frequently used molecular markers in olives. This is also evident from the large number of publications available pertaining to the use of SSRs in olive research. The characteristic features such as the multiallelic nature, wide genomic distribution, codominant inheritance, locus specificity, high mutation rates, utility as functional markers (present in transcribed regions), cross-transferability, amenability to automation, easy in silico mining and primer design have established SSRs as the markers of choice in most species [9,10]. Detailed reviews are already available, explaining the development, uses and advantages of SSR markers in plants [11,12,13], and these can be consulted for more elaborate information.

In olives, microsatellite markers have been used in various applications such as cultivar identification, characterization of autochthonous olives (ancient olive trees and oleasters), the management of olive germplasm banks, phylogenetics, diversity analysis and mapping. Moreover, these have also been widely utilized in the authentication and traceability of cultivars in olive agri-food products. Most of the studies involved the use of nuclear genomic SSRs, and recently expressed sequence tag (EST)-based SSRs or the EST-SSRs are also being exploited in several olive genetic studies. Olive SSRs have also been used in combination with other marker systems such as amplified fragment length polymorphisms (AFLPs), inter simple sequence repeats (ISSRs), single nucleotide polymorphisms (SNPs) and random amplified polymorphic DNA (RAPD) in various studies related to mapping, cultivar discrimination and genetic relationships [14,15,16,17,18,19,20,21,22,23,24]. Microsatellites, being so extensively applied in olive germplasm cataloging, authentication and traceability studies, need to be reviewed in detail, and therefore, the present review aims to elaborate on the development of SSRs in olives and specifically targets their use in olive cultivar identification, cataloging of germplasm and the traceability of oils and table olives. Information generated through such studies has been thoroughly compiled and presented in this review through extensive literature searching, mainly using Google (www.google.com accessed on 26 July 2021) and Google Scholar (scholar.google.com, accessed on 26 July 2021). Research articles and reviews covering a wide timeframe and encompassing information about olive distribution, the development of SSR markers and databases on olives and their vast applications were referred. Since the aim of the review is to mainly highlight the utility of SSR markers in the characterization of germplasm banks and local, wild and centennial olive germplasm, thereby leading to proper cultivar identification and cataloging and utilization of such information in olive agri-food authentication and traceability, articles pertaining to these fields were mainly included in this review. The review should be useful to researchers working in the above-mentioned areas. Key factors that affect the applicability and usefulness of microsatellites in olive varietal identification are also emphasized and discussed in the manuscript.

## 2. The Olive Germplasm

The olive (*Olea europaea* L.) belongs to the family Oleaceae, which comprises around 30 genera and over 600 species. The genus Olea has some 35 species, including both *O. europaea* subsp. *europaea* var. sativa (cultivated olive) and *Olea europaea* subsp. *europaea* var. sylvestris (wild olive or oleasters). In addition, the wild olive includes feral forms which are seedlings of the cultivated olives or the result of hybridizations between the oleasters and cultivars [25,26]. Additionally, five subspecies, namely *laperrinei* (Saharan massifs), *cuspidata* (Afro-Asiatic), *guanchica* (Canary Islands), *maroccana* (Morocco) and *cerasiformis* (Madeira), comprise the *Olea europaea* complex.

The olive was probably domesticated in the Middle East about 6000 years ago [27]. Afterward, commercial shipping spread this crop westward across the Mediterranean Basin, leading to complex genetic relationships among cultivars [4]. The empiric selection of outstanding individuals within wild olives, crosses between the previous selected or introduced cultivars and other local cultivars or wild olives in all growing areas have yielded a huge number of local cultivars. The easy vegetative propagation of the olive cultivars has allowed for maintaining the characteristics by which they were selected, such as greater productivity, fruit size, oil production and environmental adaptation. It is estimated that there are more than 2000 olive varieties worldwide [28]. The denomination of olive cultivars is usually a process synchronous to their diffusion. Initially, olive cultivars were named using generic criteria, like their outstanding morphological traits, utility of production or the locality of origin of the propagated material, or based on other characteristics [8]. Consequently, in olives, the existence of synonymy (different names for the same cultivar) and homonymy (same name for different cultivars) among and within olive-growing countries is very frequent [5,6,7,8].

Germplasm banks are facilities that permit us to ensure “ex situ” conservation of genetic resources. Clonally propagated fruit crops such as olives are typically conserved in “live collections”, which are suitable selected field plantations where the crop can fulfill its normal biological cycle [29]. Prospecting surveys of olive cultivars in many countries and the exchanges of cultivars between countries have contributed to the high number of conserved accessions in “ex situ” collections. Bartolini et al. [7,30] reviewed for the FAO the accessions conserved in approximately 100 regional and national collections in 54 countries, which include more than 4000 accessions supposedly belonging to 1250 cultivars [31]. Most of these cultivars come from major producer countries like Italy (538 cultivars), Spain (183), France (88) and Greece (52) [32]. Since 1994, the IOC has been promoting a network of banks to preserve the heritage of olive varieties grown in countries around the world. The network presently includes a total of 23 germplasm banks, housing over 1700 varieties andis composed of 3 international banks—Cordoba (Spain), Marrakech (Morocco) and Izmir (Turkey)—and 20 national banks (Albania, Algeria, Argentina, Croatia, Cyprus, Egypt, France, Greece, Iran, Israel, Italy, Jordan, Lebanon, Libya, Montenegro, State of Palestine, Portugal, Slovenia, Tunisia and Uruguay) (https://www.internationaloliveoil.org/the-ioc-network-of-germplasm-banks/, accessed on 5 February 2021). The Olive World Olive Germplasm Bank of Cordoba (Spain) (WOGBC) was established in 1970, and it is one of the largest with more than 1000 accessions from 29 countries [33,34]. The second international bank (WOGBM) was established in 2003 in Marrakech (Morocco) and contains around 560 accessions from 14 countries (mainly from the Mediterranean region) [35]. The third international bank was recently established (2017) in Izmir (Turkey), including 274 accessions [36]. The national olive banks preserve the local as well as important international cultivars.

Despite these efforts, the exploration and conservation of the genetic patrimony of olives is still incomplete. In recent years, numerous initiatives have been promoted to explore, preserve and exploit unknown material, including minority local varieties, centenary trees and wild olive populations (see Section 4.1). It is indeed very clear from the above information that a vast collection of olive cultivars is presently available, but challenges related to correct denominations, geographical origin and proper cataloging of these germplasm still persist, and molecular tools such as SSR markers can be a preferred choice for addressing these aspects, contributing to the proper authentication of agro-food products.

## 3. Microsatellites in Olives

### 3.1. Development and Available SSRs

The earliest reports of the development of microsatellites in olives are from the year 2000 by two independent groups. Rallo et al. [37] developed 13 SSR loci (prefixed as IAS-oli) by sequencing 43 clones screened as positive on a GA-enriched olive genomic library of the cultivar “Arbequina”. Among these, only five were found to be polymorphic when analyzed for polymorphism in 46 olive cultivars. The occurrence of repeats, other than the enriched “GA” repeats, was found in the form of compound microsatellites and presumed to be common in the olive genome. Sefc et al. [38] screened a size-selected olive genomic library for GA and CA repeats and designed primers (prefixed as ssrOeUA-DCA or DCA) for 28 microsatellite loci. Among these 15 loci, amplified specific products were polymorphic across a set of 47 olive trees from Iberian Peninsula and Italy. In the year 2002, other groups simultaneously reported the genomic library-based development of microsatellites in olives. Carriero et al. [39] screened a (GA/CT)_n_-enriched genomic library and characterized 20 SSR primer pairs (prefixed as GAPU) in 6 olive cultivars and finally reported 10 polymorphic SSR loci after testing on a set of 20 olive accessions. An average of 5.7 alleles per SSR loci was obtained with these markers. Although enriched for dinucleotide repeats, clones in the library also possessed “CCT” and “TTC” trinucleotide repeat motifs. Cipriani et al. [40] also reported the selection and sequencing of 52 SSRs from (AC/GT) and (AG/CT) repeat-enriched genomic libraries of the olive cultivar “Frantoio”. Out of these, a set of 30 SSR primers (prefixed as UDO99) were screened for polymorphism in 13 olive Italian cultivars. GA and CA repeat-enriched libraries were also developed by De La Rosa et al. [41] from the cultivar “Picual” and designed 13 primer pairs (EMO prefixed), out of which only 6 were found to be polymorphic in a set of 23 olive cultivars and were also tested for cross-species transferability.

To further expand the arsenal, the olive cultivar “Arbequina” was used in genomic library preparation and enrichment for GA, GT and ACT repeats by Diaz et al. [42]. However, inserts with the “ACT” repeat motif were not obtained even after the enrichment step. Specific primers (prefixed as IAS-oli) could be designed from 10 of the sequences containing repeats and an additional 14 sequences available from an earlier report. Gil et al. [43] also employed similar techniques of genomic library enrichment, screening and sequencing with the olive cultivar “Lezzo”, and they reported 12 polymorphic SSR primers (prefixed as ssrOeIGP) when amplified in a set of 33 olive cultivars. All these genomic SSRs have been extensively used in the characterization of olive cultivars and molecular genetic studies in olives, as reviewed in the sections below. Series DCA-, GAPU- and UDO have been very used; nevertheless, others (e.g., the EMO and IAS-oli series) have been scarcely used. Most of these attempts involved dinucleotide repeat-containing sequences for the primer design, and the GA/CT motif was commonly used. An olive genome is presumed to have a relatively frequent occurrence of compound microsatellite motifs, as found in most of the SSR development studies described above. Multiple amplification products were also reported in some genotyping experiments and probably occurred due to, for example, priming at more than one site, ploidy of the species, the presence of compound microsatellites and genome duplication events [37,40].

EST-SSRs have gained interest in recent years, owing to their easy development through user-friendly bioinformatics tools, higher cross-transferability across species and ability to be used as functional markers in marker-assisted breeding [10]. With the beginning of sequencing projects and advanced sequencing technologies, genomic resources in the form of whole genome sequences and transcriptomes have been made available in public databases for olives. These are a rich source for the in silico development of SSR markers in olives. The availability of different transcriptomes has given researchers the opportunity to screen and design primers for microsatellite repeats present in the coding regions of the genome, thus allowing association of marker variability with phenotypic traits in olives. Data from cDNA libraries sequenced as a part of the OLEAGEN project, an olive genomic project in Spain [44] was used to extract sequences with core hexanucleotide repeats, and a set of eight EST SSR primers were designed (prefixed as OLEAGEN-H) which were successfully tested for genotyping as well as paternity testing in olives and were found to be comparable to dinucleotide-based genomic SSRs reported in earlier studies [45].

Adawy et al. [46] identified 8295 SSR repeat motifs after in silico mining of the EST sequences available in the NCBI database and described 1801 EST SSR primers (prefixed as Oe-ESSR) that could be amplified in different genes. Among the set of ESTs, the highest percentage (77.6%) for mononucleotide repeats and lowest for tetranucleotide repeats (0.29%) were reported, with the AAG/CTT repeat dominating among trinucleotide types and AG/CT dominant in the dinucleotide repeats. Twenty-five primers randomly chosen for amplification in a set of 9 cultivars were able to amplify, and 10 of these were found to be polymorphic. Tissue-specific transcriptomes [47,48,49] were utilized for the in silico mining of microsatellite repeats in transcripts in [50]. Trinucleotide and longer repeat motifs containing sequences were BLAST aligned to available olive genome data (oleagenome.org), and after screening for locus redundancy, 80 SSR sequences were targeted for primer design. From a prescreening of 5 olive cultivars for amplifiable loci and expected product size, a set of 26 EST SSRs were finalized (prefixed as OLEST). The authors described a set of the 10 best OLEST SSRs after allele sequencing and validation on a larger set of olive cultivars and related species as potential functional markers in olives. EST SSRs (prefixed as OeUP) were also identified in [51] from a transcriptome of developing fruits of the olive variety “Istrska belica” [52]. Dinucleotide repeats appeared to be abundantly present (36%), with “GA” as a common repeat motif and trinucleotides showing a presence of 33% and “GAA” as a common motif. Out of the 110 EST SSRs chosen for primer designing, 46 showed positive amplification and polymorphism when validated on a set of 8 cultivars and analyzed for diversity among 24 olive varieties. A final set of 27 EST SSRs was recommended on the basis of a low null allele frequency and no deviation from the Hardy–Weinberg equilibrium for diversity and population genetics in olives. Dervishi et al. [53] also performed in silico mining of developing fruit transcriptome of the variety “Istrska belica” for tri- and tetranucleotide repeats and reported 12 primers (prefixed SNB and SiBi) out of 35 EST SSRs for olive genetic studies. Gene annotation for sequences carrying microsatellite repeats was also performed, and genes for disease resistance were reported. Similar to earlier reports, the “AAG” motif was found to be most prevalent among the trinucleotide repeats which were found in 0.18% of the sequences. In the case of tetranucleotides, “AAAT” was most frequent, and the number of repeat units in a sequence ranged from 6 to 21 in the case of trinucleotides and 4–14 for the tetranucleotides. SSRs were also found to exist in compound form in a few of the cases.

More recently, genomic SSRs based on trinucleotide repeats (with at least five core repeats) were retrieved from the whole genome sequence information in olives, and SSR primers were developed (prefixed as BFU), covering most of the chromosomes. Twenty-one SSRs were found to be highly polymorphic and effectively discriminated among a panel of 53 accessions of olives [54]. EST SSRs have also been developed by Gómez-Rodríguez et al. [55], where tetra-, penta- and hexa-nucleotide repeats were retrieved from cDNA sequences, and primers were designed (prefixed as Olea). These newly developed markers could successfully discriminate the cultivars present in the core collection of olives available at the Worldwide Olive Germplasm Bank of Cordoba, Spain. Moreover, both the genomic and EST SSRs in olives have shown transferability across oleasters as well as cultivated olives [41,53,56]. Table 1 depicts the key genetic indices as observed while developing different microsatellite resources in olives. These SSRs are a valuable resource and can be utilized in various studies related to germplasm characterization, cataloging, cultivar identification and authetication in food products as discussed in the sections below.

### 3.2. SSR Protocols for Cultivar Genotyping

Allele size discrepancies found while comparing the same set of SSRs across different samples and laboratories make the task of fingerprinting cultivars quite challenging, and thus, the utility of SSRs in cultivar authentication or in food traceability is also hampered. SSR protocols for the genotyping of olive cultivars and consensus sets of microsatellites have been proposed by various research groups for uniform data analysis and comparison. With an aim to standardize a set of SSR markers for olive genotyping, Doveri et al. [57] found that among 17 SSR markers, 6 (DCA3, DCA8, DCA11, DCA13, DCA14 and DCA15) showed maximum concordance between data points scored from all partner laboratories. Emphasis was made toward harmonization of SSR profiles for better resolution of the alleles. Baldoni et al. [58] performed an exhaustive exercise across four independent laboratories and proposed a consensus set of 11 SSRs (UDO-043, DCA9, GAPU103A, DCA18, DCA16, GAPU101, DCA3, GAPU71B, DCA5, DCA14 and EMO90) for olive genetic studies. SSRs were ranked according to the peak intensity, stuttering, null alleles, number of amplified loci and allelic error rate, which were calculated to determine the concordance of the SSRs being tested. Allelic ladders were constructed using a set of genotypes which carried true-sized alleles as confirmed by sequencing to identify the corresponding alleles between labs and to reduce the chance of mistyping alleles. The generation of allelic ladders using known profiled cultivars will allow univocal allele binning and assigning correct sizes to the new alleles. The SSRs present in the consensus list have been used in several genotyping and diversity studies of olives since then.

A protocol was also proposed by Trujillo et al. [8] using a nested set of 5, 10 and 17 SSR markers that allowed for quick characterization, authentication and identification of olive cultivars present in the WOGB in Cordoba, Spain and which could be used for management of germplasm resources in any olive gene banks. A molecular key for the identification of cultivars was also proposed by Aksehirli-Pakyurek et al. [59], where a classification binary tree (CBT) was developed and provided sorting of unknown new material that could be originating from any of the cultivars being analyzed. Hence, well-accepted SSR allelic profiles for specific cultivars are absolutely essential in order to avoid any confusion during molecular genotyping by different laboratories. This will also help in adopting a more uniform and application-worthy traceability and authenticity protocol based on SSRs.

### 3.3. Genotyping Methods

Over the years, genotyping methods used for SSR analysis have advanced to a great extent. When the aim is to specifically use SSRs for food authenticity and traceability, the genotyping methods being used are of the utmost importance, as any discrepancy in allele identification may lead to wrong cultivar identification and hamper the results. Earlier research mainly involved the use of agarose gel electrophoretic separation of SSR amplification products, and the resolution of alleles with 2–4 bp (base pair) differences in size was quite difficult. Denaturing polyacrylamide gels (4–8%) were also used for fragment separation [39,40,60], as these allow for better resolution compared with agarose gels when small base pair differences are to be identified, but these are more cumbersome to prepare, use toxic chemicals like acrylamide and involve silver staining for visualization of the separated bands. Development of more precise separation matrices in the form of high-resolution agarose have been used in amplicon separation in olive SSR analyses to resolve amplicons that differ in size by as little as 2% [37]. With more and more advancement in amplicon resolution and separation methods, matrices such as polyacrylamide and agarose are becoming obsolete and being replaced with automated capillary electrophoresis techniques and sequencing-based instruments which could achieve more sensitive allele separation and base pair calling. These advanced technologies reduced the separation time; hence, results could be obtained faster, and working with a huge sample size became easier. Moreover, integrated data analysis software, multiplexing, better reproducibility and elimination of staining procedures makes automated sequencers quite advantageous over the conventional methods of genotyping. This becomes very important when SSRs are to be used as a potential tool in olive authentication and traceability. Robust allele separation and detection is very crucial in such cases and thus requires high-throughput techniques. One of the major limitations while using microsatellites is the allele calling differences that may emerge due to polymerase slippage, DNA quantity or quality and the use of different instruments and reagents by different laboratories. Additionally, variations in results may arise due to post-PCR handling of samples in the case of gel-based platforms. These factors may cause problems in accurate determination of cultivar-specific SSR profiles and hence need to be taken into consideration while comparing genotyping results across laboratories and identifying correct cultivars [58].

High-resolution melting (HRM) analysis, an advanced method that compares the melting curve profiles of double-stranded DNA products and detects polymorphism, has recently been used as an alternative to gel-based polymorphism detection methods in olives and other species [61,62]. HRM shows greater resolving power compared with conventional melting curves, which are based on only the value of the melting temperature (Tm) and may not give better discrimination between different genotypes [63,64]. More nucleotide variations associated with the flanking regions of repeat sequences, such as single-nucleotide polymorphisms (SNPs), can be detected through this method and hence expand the applicability and potential of SSR marker systems. Refinements in the method are still going on so as to overcome challenges like specificity of the PCR, multilocus markers, and a high number of alleles [64]. Thus, continuous advancements are being made toward achieving more effective and accurate genotyping of the samples. This would help adopt a uniform method for olive genotyping, and hence information could be easily communicated and transferred between laboratories.

### 3.4. SSR Databases

It is indeed very clear that large-scale SSR genotyping projects have generated a vast amount of molecular data for different cultivars across the olive-growing regions of world. Nevertheless, this remains unutilized and inaccessible most of the time. A database is a necessary tool to correctly catalog any germplasm bank and optimize its management. Moreover, the database is the keystone to guarantee that a commercial edible product (oils or table olives) matches the cultivar specified on the label. For these reasons, the data from such independent studies need to be available on uniform platforms for easy access and use of the information. Attempts have been made to develop informative databases for olive trees, such as the Istrian olive database (http://old.iptpo.hr/iod, accessed on 20 January 2021), formed by assembling information about the morphological and molecular profiles of Istrian olive cultivars. This was an outcome of the DNA fingerprinting study of olive varieties of Istria conducted by Poljuha et al. [65]. The OLEA database (http://www.oleadb.it/, accessed on 20 January 2021) was yet another olive molecular database established in 2007 by researchers in Italy, and it comprised SSR marker data of a broad set of olive cultivars. Users could search for cultivars corresponding to a particular data type and variety identity and also look for cultivar information across different olive collection facilities.

With the generation of more and more EST information in public databases and the development of EST SSRs in olives, genetic studies have also been conducted using these SSRs. ReprOlive (http://reprolive.eez.csic.es, accessed on 20 January 2021) is a freely available database that gives access to the reproductive transcriptomes of olive trees, where information can be retrieved about tentative transcripts containing SSR units and suitable primers can be designed [66]. Another comprehensive olive database, the Olive Genetics Diversity Database (OGDD) pertaining to SSR molecular data, was generated by Ben Ayed et al. [67], and it is reported to contain morphological, chemical as well as molecular genetic (SSR) information about several olive varieties and oils. However, it is emphasized that the regular addition of newly generated information, updated software and easy access of these databases are required so that users can access the webpages and information smoothly. Public databases would make comparative studies much easier and more useful in the identification and authentication of cultivars and their products, and the information could be used by breeders, population geneticists and researchers across laboratories.

## 4. Applications of SSRs: Cataloging of Olive Germplasm, Food Authenticity and Traceability Studies

### 4.1. Cataloging Olive Germplasm

The varietal cataloging process implies (1) characterization or description of the cultivars at different levels (e.g., morphological, molecular or agronomical); (2) identification, a process that allows us to classify or differentiate one cultivar from the rest; (3) authentication, a process that guarantees that a cultivar corresponds to the original cultivar from its natural area of cultivation or origin; and (4) assigning the correct name to the cultivar once identified and authenticated and defining its synonyms and homonyms [68]. Therefore, the cataloging of any bank should be an essential requisite before using plant material for conservation, propagation and breeding purposes. Varietal information is also a key identifier in quality control for high-value virgin olive oils and table olives in the food industry.

**Figure 1:**
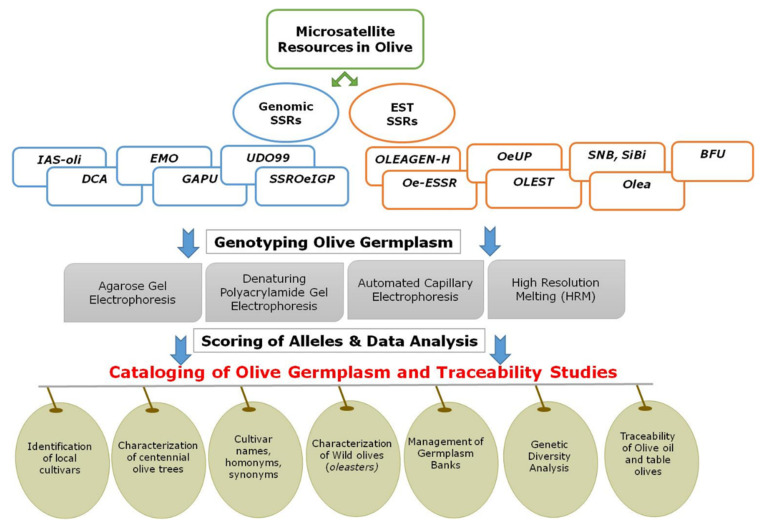
Microsatellite resources and applications in olive germplasm cataloging, authenticity and traceability.

In species like the olive, this task becomes particularly challenging. There are several factors that contribute to this, such as the vast number of olive cultivars, the use of generic criteria to name them and the misunderstanding around basic concepts that has led to a confusing scenario. In addition, the heterogeneity of criteria and methodologies applied for cataloging has hampered the completion of varietal cataloging in most traditional olive-growing countries. In this regard, the integration of molecular markers, particularly the microsatellites with the pomological scheme defined by Barranco et al. [69], has allowed for important advancements in the cataloging of olive germplasm [8]. In this work, the challenges of the incorporation of SSR markers both for the cataloging of germplasm and traceability studies in olive oil and table olives are highlighted. Figure 1 summarizes the microsatellites available in olives, the genotyping process and their applications in the cataloging and management of olive germplasm.

#### 4.1.1. Cataloging of Germplasm Banks

Collections in a germplasm bank are a proper source of confirming the true identity of the cultivar in question. Hence, proper identification and cataloging of plant material becomes a prerequisite for efficient management of germplasm banks. The cataloging (characterization, identification, authentication and naming of the cultivar) of the accessions of any olive germplasm bank should be compulsory before distribution of any plant material from that bank. Only the diffusion of true-to-type cultivars will avoid worldwide confusion between the denominations and cultivars existing in almost any world germplasm collection [30]. Aside from that, the SSR profiles of correctly identified and authenticated material can be used as a reference when dealing with the authenticity and traceability of olive products. In this direction, Trujillo et al. [8] exhaustively characterized, identified and authenticated the 499 accessions (824 trees in total) present in the WOGBC in Córdoba, Spain, representing samples from 21 countries using both phenotypic characters and molecular profiles generated by 33 available SSR markers. Several cases of synonyms and homonyms were detected and rectified, along with the identification of unique genotypes. The WOGBC has now become one of the most characterized olive germplasm banks and has paved way for other worldwide collections to also be well cataloged.

Trujillo et al. [68] also proposed and presented a guide in the international seminar “The IOC Network of Germplasm Banks and The True Healthy Olive Cultivars Project” held in Cordoba (Spain) in 2019. In this guide, the successive necessary steps and methodologies for accomplishing these goals are described, from the arrival of the vegetal material to the bank to the establishment of the plants in the field collection once identified, authenticated and free of pathogens. The molecular protocol is based on a set of 17 previously selected SSRs. All of them are robust and extremely polymorphic, with almost a limitless capability to catalog olive cultivars [8]. Aside from that, in most of the IOC Network collections, there is a considerable amount of information generated with SSR markers. These exhaustive studies establish the potential of microsatellites as robust markers for the characterization and identification of cultivars in rich olive germplasm. Better management of ex situ collections would in turn facilitate the easy exchange of germplasm material even at international levels, eliminate any mislabeling or misinterpretation of cultivars and ensure a reliable supply of cultivars to research labs, breeders and markets. These are indeed very useful resources in developing olive authentication and traceability studies, where the genotypic profile of any cultivar in question can essentially be matched with its true representative maintained in these worldwide collections.

#### 4.1.2. Local Cultivars and Centennial Trees

In the last 25 years, important socioeconomic changes in many Mediterranean countries have driven significant technological improvements in olive cultivation. These changes are increasing the risk of genetic erosion of olive germplasm because local traditional cultivars are being replaced by a few cultivars that are suitable for new mechanically harvested plantations. Therefore, the identification and conservation of traditional olive cultivars are currently high-priority tasks that are needed to ensure the sustainable use of those cultivars in the future [70]. Microsatellite markers have been proven to be immensely useful in describing olive cultivars cultivated locally in certain regions [71,72,73,74,75,76]. Genotypic data about these local cultivars are useful information when authenticating commercial products coming out of these areas and certifying the origins of cultivars.

In Montenegro, when characterized using 10 SSR markers from the consensus set described by Baldoni et al. [58], the genotypic profile of the oldest olive tree, “Stara Maslina”, was found to be quite distinct from other ancient trees and main varieties, including the most diffused “Zutica Bar” variety. In addition, all locally grown and ancient germplasm of Montenegro were grouped together into a separate cluster when analyzed with other foreign cultivars [77]. Similarly, the autochthonous olive germplasm in Crete, Greece, represented by three cultivars (“Koroneiki”, “Mastoidis” and “Throubolia”) were characterized, along with two cultivars from Turkey and some representative wild genotypes from Crete, using seven informative SSR markers (from the DCA, UDO99 and IAS-oli series). The autochthonous cultivars were grouped into separate clusters showing their distinctness, and the cultivar “Throubolia” was found to be close to Turkish cultivars, indicating possible exchange or movement of the germplasm in the past [59]. Such studies supported by SSR-based genotypic information highlight the uniqueness of local germplasm and point toward more targeted genetic evaluation and conservation of such germplasm in olive-growing regions. Additionally, the information thus generated can also be utilized in developing SSR-based cultivar identification keys to be used in any future authentication of agri-food products based on such cultivars.

Since antiquity, olives have been grown and cultivated in the Mediterranean region of the world, and to date, many such centennial olive trees can be found growing in different regions. Microsatellites have been the molecular marker of choice for the characterization and identification of monumental or centennial olives from different olive-growing regions and proved helpful in generating valuable information with respect to the genotypic identities of trees. These studies supported the hypothesis that ancient olive trees might be unknown traditional cultivars that remained uncharacterized. Rotondi et al. [78] reported that most of the 206 ancient olive trees growing across the Emilia-Romagna region in Italy belonged to 10 cultivars that were already characterized, and the origins of the remaining genotypes remained unknown. In yet another study, 4526 ancient olive trees were surveyed in the “Taula del Sénia” (M-TdS) area (northeast Iberian Peninsula), and a subset of 293 trees was molecularly characterized using eight SSR markers, which revealed 43 different genotypic profiles, with 98% of the trees belonging to the local cultivar “Farga” [79]. Erre et al. [80] genotyped 21 wild and 57 cultivated olives in Sardinia using 13 SSR markers, where novel genotypes were identified and cluster analysis grouped the trees into distinct “wild” and “local” gene pools. Hence, valuable information could be deciphered with reference to the cultivar identity and existence of these trees using molecular as well as phenotypic tools. This would also be helpful in devising strategies for the cataloging, conservation and protection of such a rich ancient resource. The molecular information in the form of SSR profiles generated through such genetic studies can be very useful in cases where local cultivars are blended with commercial ones or when any high-value local cultivars are being adulterated.

#### 4.1.3. Characterization of Wild Olive Populations

Wild germplasm in olives, also known as oleasters, can be exploited in breeding and genetic improvement programs as a rich source of variation in the development of varieties with improved traits, such as biotic and abiotic resistance and increased growth and yields. Microsatellite marker-based diversity studies and the estimation of genetic relationships within wild olive populations and between cultivated and wild forms were reported [80,81,82,83,84,85,86,87,88,89,90]. This would give better insights into the history of the domestication of olives, the regional distribution of genetic diversity and any gene flow between oleasters, feral forms and cultivated types. This topic has been recently reviewed by Belaj et al. [91]. Therefore, to obtain more detailed information, the reader is referred to this review.

In summary, Table 2 provides a list of studies highlighting the various applications of microsatellites in the characterization of olive genetic resources. These studies actually provide useful information about the various microsatellite markers used, and the different genetic indices thus generated can help in the selection of the most appropriate set of SSRs for any future work related to characterization or cultivar authentication. High genetic variability can be utilized in selecting superior genotypes and cultivars for future breeding programs and cultivation. Broad genetic diversity in olive germplasm is also reflected by high heterozygosity levels (both expected and observed heterozygosity) obtained through SSR analysis. For the most part, the expected heterozygosity (H_e_) values were lower than the observed heterozygosity (H_o_) in olives, as represented in Table 2. Another informative genetic index for SSR usefulness is the polymorphic information content value (PIC value), which in the case of olive SSRs was >0.5 in most of the cases for different SSR loci and reported to be as high as 0.95 by Omrani-Sabbaghi et al. [92].

### 4.2. Agri-Food Traceability: Olive Oil and Table Olives

Two of the essential components of Mediterranean cuisine, table olives and olive oil, are among the most valuable agri-food products, especially in European markets. Their rich nutritional value and antioxidant properties have also attracted customers from non-olive oil producing areas such as the U.S. and Asia. Increasing demands and market value have tempted certain groups toward fraud and adulteration of high-quality extra virgin olive oils as well as table olives, with the mixing of cheaper low-quality oils such as other vegetable oils [105] and mislabeling of products produced from high-value cultivars or olive-growing regions being among the identified adulterations [106]. To prevent such fraudulent practices, the European Union (EU) has enacted regulations and introduced certifications (European Council Regulation EEC/2081/1992) in the form of “protected designation of origin” (PDO) and “protected geographical indication” (PGI) and launched a consortium-led project called “OLIV-TRACK” to work on olive oil traceability. Additionally, recent projects such as the OLEUM project (http://www.oleumproject.eu/, accessed 20 June 2021) and the Food Integrity Project (https://secure.fera.defra.gov.uk/foodintegrity/index.cfm?sectionid=21, accessed 20 June 2021) have also presented strategies to tackle olive oil fraud. Apart from the geographical origin of the cultivar and processing methods, the cultivar genotype is one of the key determinants in defining these designations. Therefore, methods are needed that can ascertain the authenticity of cultivars present in a particular batch of olive oil. The concept of cultivar authentication has primarily been used in the context of modern food technology to guarantee that the commercial edible product matches the cultivar specified on the label [107,108,109]. The authenticity of olive oil and table olives has been assessed through conventional methods, including chemical analyses where the presence of the key metabolites responsible for a peculiar flavor and taste is examined. These mainly include the HPLC-based identification of distinct metabolites [110,111]. Several other techniques such as lipid profiling, stable isotope analysis and chromatography-based metabolite analysis have also been used in olive product authentication and traceability [112,113]. Although these methods allow the identification of cultivars and their origins, complex multivariate analyses and statistical procedures are generally needed, which makes these challenging tasks. Additionally, many of these methods are affected by the environment and physiological conditions during the growth of plants, and hence variations in compositions may be seen. Therefore, DNA-based detection methods have gained interest in recent years, as DNA is unaffected by environmental conditions, and thus more specific, accurate and sensitive results could be obtained regarding the origin and identity of a cultivar. Various DNA-based molecular markers have been used in the authentication of olive trees and oil [114,115]. As already detailed in this review, SSRs possess a high power of discrimination and are among the most widely employed molecular marker systems in olives. Difference in SSR profiles between olive oil-producing cultivars can be used to identify their presence in monovarietal oils as well as mixtures of olive oils. The isolation of DNA in adequate amounts and quality from difficult matrices like olive oil is a challenging task, and the success and reproducibility of PCR amplification and marker analysis largely depends on this. Over the last decade, different isolation protocols and kits have been tested and modified for better DNA extraction from fruits and oils, and these studies highlight the importance of DNA quality and its impact on molecular marker-based tests [116,117,118,119,120]. Recently, Piarulli et al. [121] compared four DNA isolation methods referenced in the literature and came up with a modified method based on the work of Consolandi et al. [122] for the extraction of DNA from extra virgin olive oil in a much smaller time frame (4 h as compared with the 30 h reported) and involving low-cost options. A washable and reusable miniaturized device has been developed as well and tested for highly efficient DNA purification from olive oil, providing an increased surface-area-to-volume ratio when compared with other approaches, allowing highly efficient DNA purification and concentration from samples with minute DNA contents [123]. Molecular markers that amplify shorter fragments are supposed to work efficiently with low-quality or fragmented DNA isolated from oil, and SSRs and SNPs are the favored choice in such cases. Here, key achievements in the field of olive oil and table olive traceability using microsatellite or SSR markers are reviewed and summarized in Table 3 with details of the SSR markers and sample types used.

Breton et al. [116] used magnetic beads for DNA purification and amplified SSR alleles from leaves as well oil DNA. The SSR patterns were verified in virgin oil samples of known origins, either in separate cultivars or in mixtures, as well as in commercial virgin oil samples available from markets. Virgin olive oil originating from 10 different olive cultivars were also identified by Pasqualone et al. [124], and a set of three primers (DCA4, DCA17 and GAPU89) was used to describe an identification key for olive cultivars and oil traceability. Testolin and Lain [117] reported DNA extraction from olive oil, comparing different protocols and commercial kits and utilizing conventional and nested SSR-PCR to identify specific cultivar DNA in oil. Similarly, Muzzalupo et al. [118] performed SSR-based authentication of virgin olive oil from “Ogliarola salentina” and Pasqualone et al. [125] identified a PDO-designated extra virgin olive oil (Collina di Brindisi) which contained aminimum of 70% oil from the cultivar “Ogliarola salentina”.These studies established the utility of microsatellites in authenticating a cultivar in a mixture of oils as well.

The use of principal component analysis (PCA) was emphasized in a study with 23 olive oil samples of Portuguese origin (11 monovarietal and 12 commercial oils), which were fingerprinted using 4 SSR loci in combination with 2 RAPD and 4 ISSR markers. No correlation among the common denominations was revealed and commercial samples from the same olive oil brand as well as the samples from the PDO olive oil Tras-os-Montes were found to be distributed in different PCA quadrants. The use of a larger set of markers was therefore required in order to cluster the cultivars and identify each denomination. The study found PCA analysis to be useful in the categorization of samples according to the regions of origin [126]. While dealing with oil traceability through genetic markers, the presence of alleles from pollinators also needs to be distinguished in order to infer denominations correctly, as observed by Ben-Ayed et al. [119] and Alba et al. [127], where parental contributions are assessed while comparing the microsatellite profiles generated from the DNA of the leaves and oil of certain cultivars. The utility of microsatellites in the genetic traceability of oil in agro-food chains was also established when DNA isolated from the drupes or leaves samples of three olive oil cultivars, namely “Pisciottana”, “Frantoio” and “Leccino”, were genotyped using six SSRs, and similar genetic profiles were obtained with their monovarietal oils. A 1:1 DNA mixture from two extra virgin monovarietal oils was also tested and could detect the expected alleles in the mixture [99].

**Figure 2:**
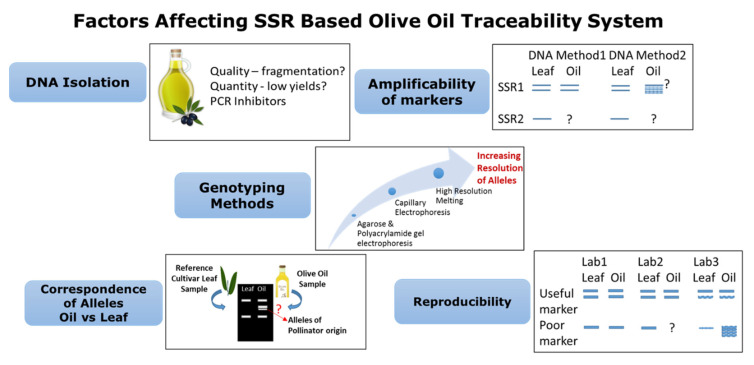
Process and main factors influencing the applicability of a microsatellite marker in the authentication and traceability of olive oils and table olives.

Microsatellites have also been used in traceability analyses for PDO table olives. Three Italian PDO olives could be reliably identified among a set of 10 olive cultivars using 16 SSR primer pairs. A power of discrimination as high as 0.9 was obtained in the microsatellite set used for analysis [130]. A combination of genetic and biochemical tools in olive oil traceability studies can add to the accuracy of the experiments. Correlation between the SSR genetic data from cultivars and chemical and sensory profiles of nine monovarietal oils was observed by Rotondi et al. [137]. However, no correlation was obtained between genetic and pleasant flavor profiles. A bunch of parameters could play a role in the success of a traceability system based on genetic markers like microsatellites. An evaluation of such parameters was conducted by Vietina et al. [128] through the genotyping of 21 monovarietal oils obtained from 16 cultivars using 11 microsatellite markers. Each marker was assessed for its amplification ability over different oil DNA, reproducibility across a set of replicates in an experiment and correspondence of alleles in oil as well as leaf DNA. Significant correlation was found between the amplification ability and DNA yield, indicating the role of the extraction method. SSR marker GAPU89 gave a total correspondence and amplification ability value of 49.32%, and marker DCA5 was found to have the highest reproducibility, being 71.43 ± 21.82%. The high standard deviation values were attributed to variations within the samples caused by DNA extraction. Microsatellites were also successfully used by Ben-Ayed et al. [129] in the authenticity and traceability of virgin olive oils, and they also reported the non-correspondence of SSR profiles between oil and leaf DNA in some cases, thereby further strengthening the importance of distinguishing the pollinator and maternal alleles. Figure 2 summarizes the process and the main factors that may potentially affect the molecular traceability of olive oils and table olives when using SSR markers. As depicted in the figure, during DNA isolation, DNA that is too fragmented and very low yields may not always provide sufficient target templates and hence do not amplify the correct alleles. Similarly, the presence of inhibitor compounds from DNA extracts may lead to poor PCR amplification. Amplificability of the markers is also required to be checked for different SSRs in DNA isolated by different methods. Only those markers which give a consistent result in one or two methods should be used further. For the genotyping methods, the resolution of alleles needs to be highly precise for using SSRs in traceability and authenticity testing. Methods like capillary electrophoresis and high-resolution melting have proven to be useful. The correspondence of alleles is yet another important factor, where any microsatellite that generate similar profiles in a target oil and corresponding leaf sample of the cultivar in question can be used as a traceability marker. Ideally, the allelic pattern should be similar, but knowledge of the pollinating behavior of the cultivar is beneficial for result interpretation. As for reproducibility, an ideal SSR used for traceability should be highly reproducible irrespective of the laboratories, instruments and reagents used.

Concerns with respect to the presence of traces of pollinator DNA in extractions made out of oil matrices leading to differences in the allelic profiling of oil and leaf samples also attracted researchers toward the applicability of plastid-based markers. However, chloroplast DNA (cpDNA) among cultivars has shown low levels of variation, which has limited its use in authenticity testing or traceability analyses.

Pérez-Jiménez et al. [131] utilized nine cpDNA loci that consisted of microsatellites and small insertion–deletions (indels) to identify the olive cultivar in leaves and corresponding oil DNA. Six haplotypes could be fingerprinted, and a rare haplotype was identified in genotypes producing regionally high-valued commercial oil. The available olive plastid genome can therefore be analyzed for the presence of more such microsatellite regions. In order to overcome the challenges of DNA isolation from oil matrices, Muzzalupo et al. [132] reported a direct DNA amplification method which avoided the routine extraction step and instead used KAPA3G plant DNA polymerase (an engineered DNA polymerase which could tolerate plant PCR inhibitors) for SSR amplification of membrane-filtered DNA molecules. DNA isolated from this method was used to check the traceability of three distinct types of virgin olive oil. The diagnostics power of microsatellite markers was further proven in the analysis of processed olives by Crawford et al. [138], where a panel of 5 SSRs was selected out of the 15 tested to authenticate California-style olive cultivars, widely marketed as packed forms. Based on the differences in allele combinations generated through these markers, any two samples could be differentiated. While comparing the genotyping method based on SSR alongside fatty acid analysis, phenolic content and nuclear magnetic resonance (NMR) analysis, Crawford et al. [139] found NMR to be able to discriminate all four tested cultivars in their processed forms. However, the five SSR markers could still detect genetic similarity between Sevillano and Gordal cultivars and indicated possible synonymy between the two.

More recently, techniques like high-resolution melting (HRM) have been reported to be coupled to SSR genotyping for the identification of target cultivars in commercial olive oil samples. HRM gives an additional advantage of closed-tube analysis post-PCR and is a sensitive and cost-effective method. Montemurro et al. [133] identified the constituent cultivars of PDO, designated “Terra di Bari” extra virgin olive oil, using HRM curve analysis of the SSR marker DCA18, and Gomes et al. [135] also applied this method for varietal identification in monovarietal PDO as well as blended olive oils using three SSRs from the UDO99 series (UDO99-011, UDO99-039 and UDO99-024) and one SSR from the DCA series (ssrOeUA-DCA16). In addition, Pasqualone et al. [134] evaluated the effect of talc addition during olive oil processing on DNA by comparing the SSR-HRM profiles of treated as well as control samples. Similarly, Pasqualone et al. [136] carried out varietal authentication in samples from crude olive pomace oil and corresponding virgin olive oil. Chedid et al. [140] performed both SSR-HRM and SNP-HRM for authentication and trace adulteration in olive oils and found that the discrimination power of SSRs was greater in the case of monovarietal olive oils, while SNPs were the marker of choice when the oils were blended together or adulterated.

Overall, microsatellites present a desirable system for formulating olive oil and table olive traceability studies, and key parameters like DNA extraction efficiency, reproducibility of the SSR profiles, knowledge about the breeding and pollinating behavior of the cultivars in question and correspondence levels between the oil and reference leaf SSR profiles should be focused on in order to utilize the method as a successful detection tool.

## 5. Concluding Remarks

A vast amount of genetic information about olive populations, wild relatives, local cultivars and germplasm banks around the world is now available to researchers, which can be utilized for developing cultivar breeding programs and better management of global olive genetic resources. However, organizing this valuable information in the form of easy-to-access and routinely updated databases is essential for the smooth transfer and sharing of scientific knowledge to the olive research community and control laboratories for the olive industry. Olives and olive oil have been an essential part of the diets for many populations, especially the Mediterranean region, with a notable presence nowadays in the non-olive growing nations of the world as well. Therefore, genetic characterization of the available unexplored germplasm is an important step for the introduction of new and improved cultivars. There are challenges associated with use of SSRs as tools to identify olive cultivars and obtain reproducible DNA profiles extracted from its oils.

One of the main limitations in implementing a traceability system based on microsatellites or any of the marker systems is the reproducibility of genotypic profiles across different laboratories. There can be variations due to the quality of the DNA extracted and the genotyping method used, and therefore, results need to be carefully interpreted while using the same set of cultivars and markers under different conditions. Additionally, identifying pollinator origin alleles while comparing olive oil and corresponding leaf DNA is crucial for correct result interpretation. A set of reference cultivars and their respective SSR profiles should be defined globally, and this can be used as a set of controls during experiments by all the laboratories working in cultivar identification and traceability of oil and table olives in order to maintain the authenticity of the data. Olive oil and table olive quality and authenticity is a topic of concern nowadays, and continuous efforts are being made to develop traceability tools based on chemical as well as molecular methods. The available literature indicates that microsatellites are a potential marker system with excellent utility in cultivar identification and coupling with high-throughput platforms, like automated sequencers, and high-resolution melting provides much faster and more sensitive and accurate results. As developments are being made in sophisticated techniques of genotyping, the problems associated with microsatellite profiling, such as mis-scoring of alleles or poor resolution of the electrophoresis gels, are being overcome, allowing users to obtain robust and reliable molecular profiles from samples of commercial olive oil and table olives.

With the use of next-generation sequencing (NGS) technologies in olive trees, more and more genomic information is being added and can be used as a rich source for the development of new sets of long core repeats containing microsatellite markers to overcome limitations while using dinucleotide repeat-rich SSRs. The increasing number of available genomic as well as EST SSRs will not only escalate the existing molecular arsenal but also pave the way for their application in the development of functional markers and linkage, as well as association mapping, map-based cloning and marker-assisted selection in the future, in addition to variety identification in high-quality food products such as table olives and olive oil. The use of techniques like HRM has opened new ways of analyzing microsatellites and exploring their potential beyond length polymorphisms. The development and applications of SNP markers in olives have also gained attention in recent years, but SSRs still remain a marker of choice to initiate preliminary genetic studies in a collection of cultivars, especially in resource-limited laboratories.

## Figures and Tables

**Table 1:** Key genetic indices as reported for SSR markers developed in olives using enriched genomic libraries and EST sequences.

Reference	Naming of SSR Loci (Prefixes)	Type of SSR	No. of Polymorphic SSRs Reported	No. of Cultivars Used in Characterization of SSRs	N_a_	H_o_	H_e_
[37]	IAS-oli	Genomic	05	46	3–9	-	0.460–0.710
[38]	DCA	Genomic	15	47	4–15	0.283–0.979	0.357–0.859
[41]	EMO	Genomic	13	23	6–9	0.391–0.913	0.620–0.811
[39]	GAPU	Genomic	10	20	3–9	-	-
[40]	UDO99	Genomic	28	13	1–5	-	0.000–0.770
[43]	ssrOeIGP	Genomic	12	33	2–14	0.188–0.813	0.417–0.895
[42]	IAS-oli	Genomic	12	51	1–13		
[45]	OLEAGEN-H	EST-SSR	08	15	2–7	0.380–1.000	0.490–0.850
[46]	Oe-ESSR	EST-SSR	1801; 25 of these used	09	-	-	-
[51]	OeUP	EST-SSR	46	24	2–8	0.042–1.000	0.042–0.869
[50]	OLEST	EST-SSR	26	32	2–10	0.219–0.813	0.195–0.839
[53]	SNB, SiBi	EST-SSR	-	-	-	0.357–0.932	0.294–0.790
[54]	BFU	EST-SSR	21	53	3–10	0.140–0.910	0.520–0.810
[55]	Olea	EST-SSR	08	36	4–7	0.350–0.710	0.540–0.750

(N_a_) Average number of alleles per locus. (H_o_) Observed heterozygosity. (H_e_) Expected heterozygosity or gene diversity.

**Table 2:** List of studies highlighting applications of microsatellites in the characterization of olive genetic resources.

Objective	Cultivars or Accessions	Region	No. of SSRs	H_o_	H_e_	N_a_	PIC	Reference	Key Remarks
Characterization and Identification of Olive Cultivars	19	Slovenia, Italy, France, Spain	14 (DCA-1,3,4,5,7,8,9,10, 11, 13, 14, 15, 16, 17)	0.263–1.000	NA	3–12	NA	[60]	Identification key of 19 olive varieties
Characterization and Identification of Olive Cultivars	87	Iran	16 (DCA 18, 17, 16, 15, 14, 13, 11, 10, GAPU101, 103A, 89, 71B, 72, 90)	NA	NA	NA	0.620–0.950	[92]	Intra-cultivar variation, cultivar denominations and origin investigated using SSRs
Characterization of Autochthonous Olives	44	Croatia	16 (UDO-08, 12, 19, 24, 28, 31, 39, 43, DCA3, 8, 9, 10, 14, 16, EMO2, 3)	0.273–0.932	0.499–0.910	5–20	NA	[93]	SSR-based varietal discrimination achieved
Germplasm Characterization	154	Tuscany	12 (UDO-04, 06, 09, 11, 12, 17, 19, 24, 27, 31, SIU06, 08)	0.278–0.722	0.428–0.855	3–10	NA	[94]	Homonyms and synonyms detected
Database Development and Cultivar Identification	17	Mediterranean Basin	08 (DCA3, 4, 8, 9, 11, 13, 14, 15)	NA	NA	3–12	NA	[57]	Standardization of SSR set for olive cultivar studies
Characterization and Identification of Olive Cultivars	18	Bologna, Italy	17 (DCA3,4,5,7,9,13,14,15,16,17,18, EMO90, GAPU59,101,103, UDO-24, 43)	0.500–1.000	0.431–0.841	4–10	NA	[95]	Synonyms identified and diversity in germplasm revealed
Characterization of Olive Cultivars	20	Tunisia	10 (GAPU59, 71A, 71B, 103A, UDO-03, 12, 28, 39, DCA9, 18)	0.300–0.950	0.562–0.801	3–6	NA	[96]	Cultivars broadly grouped by their end use and phenotypes
Characterization of Olive Cultivars	38	Southern Marmara region, Turkey	10 (GAPU103A, 101, UDO-06, 07, 09, 11, 12, 14, 15, 35)	NA	NA	2–5	NA	[97]	Cultivar Gemlik revealed as major olive cultivar in the region
Characterization of Olive Cultivars	51	Iran	13 (DCA3,9,16,18,11,15, UDO-43, 11, 19, 24, GAPU59, 71B, 101)	0.000–1.000	0.000–0.800	1–8	0.000–0.750	[98]	Synonyms, homonyms and intra-cultivar polymorphisms detected
Characterization and Diversity Analysis	47	Southern Italy	6 (DCA3, 4, 16, 17, UDO-31, GAPU 47)	0.700–0.890	NA	11–17	0.830–0.870	[99]	Genetic distinctness of accessions from Campania region established
Characterization of Olive cultivars	10	Turkey	7 (DCA-4, 9, 11, 16, 17, GAPU-89, UDO-14)	NA	NA	3–6	NA	[100]	Misnamings among cultivars identified
Cultivar Identification Using SSR	53	Australia	7 (9, 3, 16, 18, 5, EMO90, 30)	0.490–0.980	0.480–0.840	7–12	0.467–0.813	[101]	Samples grouped into distinct genotypes
Characterization of Olive Germplasm	561	Marrakech (OWGB collection)	17 (DCA1, 3, 4, 5, 8, 9, 11, 14, 15, 18, UDO-36, GAPU59, 71A, 71B, EMO03, 90,PA(ATT)2^*^)	0.490–0.928	0.454–0.876	5–32	0.403–0.864	[102]	Construction of two core collections
Cultivar Characterization and Diversity Analysis	26	Algeria	11 (DCA9, 18, GAPU59, 71A, 71B, 101, 103A, UDO-12, 43, 28, 39)	0.135–0.889	0.070–0.510	6–21	NA	[103]	SSR genotyping allowed unambiguous identification of all the cultivars
Cultivar characterization and Diversity Analysis	39	Colombia	10 (DCA3, 5, 9, 16, 17, 18, UDO-43, GAPU101, 103A, EMO90)	0.250–1.000	0.312–0.909	3–15	0.282–0.876	[104]	Synonyms, homonyms identified and 19 genetic profiles discriminated

(N_a_) Average number of alleles per locus. (H_o_) Observed heterozygosity. (H_e_) Expected heterozygosity or gene diversity. (PIC) Polymorphic information content.

**Table 3:** Applications of microsatellites in olive oil and table olive traceability.

Sample Type	SSR Markers Used	References
Oil samples of different origins	EMO series SSR primers	[116]
VOO (virgin olive oil) from 10 Italian olive cultivars	DCA4, 15,17; GAPU71, 89, 101; UDO03	[124]
Samples of filtered and unfiltered VOO of cv. Carolea	UDO08, 09, 12, 24, 39, 043 and respective shortened internal primers	[117]
VOO of cv. Ogliarola salentina cultivar	GAPU59, 71A 103A and UDO01, 03, 39	[118]
Samples of Collina di Brindisi PDO oil (four unfiltered and two filtered oils); samples of constituent cultivars used for preparation of the PDO mix	UDO09, 19, 25, 35, 044 and GAPU89, 101	[125]
11 monovarietal olive oil samples from Portuguese cultivars; 12 commercial olive oils	DCA1, 3, 5, and 9 along with 11 RAPD and 08 ISSR primers	[126]
Oil from 2 Tunisian olive cultivars: *Chemlali* and *Chetoui*	DCA1, 3; GAPU59, 71A, 71B and UDO12	[119]
Monovarietal oils from 7 Italian cultivars (Coratina, Picholine, Toscanina, Cima di Melfi, Frantoio, Leccino and Cellina di Nardo)	DCA3, 4, 7, 14, 15, 18; GAPU103; EMO90; EMOL and UDO43	[127]
Monovarietal extra virgin olive oil (EVOO) from cultivars Pisciottana, Frantoio and Leccino and their 1:1 mixtures	DCA 3, 4, 16, 17; UDO31 and GAPU 47	[99]
21 monovarietal olive oils from 16 cultivars	EMO30, 90; DCA5, 8, 17, 18; GAPU71B, 89; UDO09; and Shortened DCA14 and EMO30	[128]
VOO from 22 cultivars	DCA1, 3, 4; GAPU59, 71A, 71B; UDO12, 09	[129]
03 Italian PDO table olives and 07 highly diffused cultivars of table olives	16 SSR markers of DCA, GAPU, EMO and UDO99 series.	[130]
14 monovarietal and commercial olive oils	09 cpDNA markers	[131]
3 VOO samples (Frantoio, Italian PDO *Terre di Bari* and other)	GAPU59, 71A, 71B, 103A; UDO01, 03, 12, 28, 39 and DCA9, 18	[132]
“Terra di Bari” PDO EVOO; 9 Apulia region cultivars; experimental mixtures of oils	17 SSR markers of DCA, GAPU, EMO and UDO99 series; HRM analysis of DCA18	[133]
Oil from cv. Coratina	SSR-HRM analysis of DCA3, 16, 18; GAPU103A	[134]
10 monovarietal olive oils from different Portuguese PDO regions; 2 commercial EVOO olive oils	HRM analysis of 15 SSR from DCA and UDO99 series	[135]
VOO and crude olive pomace oil of cv. Coratina	HRM analysis DCA4, 9 and 14	[136]
